# Maternal and reproductive health financing in Burundi: public-sector contribution levels and trends from 2010 to 2012

**DOI:** 10.1186/s12913-015-1009-7

**Published:** 2015-10-01

**Authors:** Claire Chaumont, Carmen Muhorane, Isabelle Moreira-Burgos, Ndereye Juma, Leticia Avila-Burgos

**Affiliations:** Center for Health Systems Research, National Institute of Public Health, Universidad No. 655 Colonia Santa María Ahuacatitlán, Cerrada Los Pinos y Caminera, C.P. 62100 Cuernavaca, Morelos Mexico; Performance Based Financing Planning Unit, Ministry of Public Health and Fight Against AIDS, Rue Pierre Ngendandumwe, Bujumbura, Burundi; United Nations Population Fund, Avenue d’Uvira, IMHQ BNUB, Bujumbura, Burundi; National Reproductive Health Programme, Ministry of Public Health and Fight Against AIDS, Rue Pierre Ngendandumwe, Bujumbura, Burundi

## Abstract

**Background:**

An understanding of public financial flows to reproductive health (RH) at the country level is key to assessing the extent to which they correspond to political commitments. This is especially relevant for low-income countries facing important challenges in the area of RH. To this end, the present study analyzes public expenditure levels and trends with regards to RH in Burundi between the years 2010 to 2012, looking specifically at financing agents, health providers, and health functions.

**Methods:**

The analysis was performed using standard RH sub-account methodology. Information regarding public expenditures was gathered from national budgets, the Burundi Ministry of Public Health information system, and from other relevant public institutions.

**Results:**

Public RH expenditures in Burundi accounted for $41.163 million international dollars in 2012, which represents an increase of 16 % from 2010. In 2012, this sum represented 0.57 % of the national GDP. The share of total public health spending allocated to RH increased from 15 % in 2010 to 19 % in 2012. In terms of public agents involved in RH financing, the Ministry of Public Health proved to play the most important role. Half of all public RH spending went to primary health care clinics, while more than 70 % of this money was used for maternal health; average public RH spending per woman of childbearing age stagnated during the study period.

**Conclusions:**

The flow patterns and levels of public funds to RH in Burundi suggest that RH funding correctly reflects governmental priorities for the period between 2010 and 2012. In a context of general shrinking donor commitment, local governments have come to play a key role in ensuring the efficient use of available resources and the mobilizing of additional domestic funding. A strong and transparent financial tracking system is key to carrying out this role and making progress towards the MDG Goals and development beyond 2015.

## Background

Despite important improvements made since 1990, indicators used to track progress towards Millennium Development Goal (MDG) 5 — focused on maternal and reproductive health — still fall far short of established targets in sub-Saharan Africa [1–4]. The most recent estimates from the Global Burden of Disease project show a decline in the global maternal mortality ratio (MMR) from 283.2 to 209.1 deaths per 100,000 live births between 1990 and 2013. While progress is evident, these numbers align with previous studies in concluding that most countries will not reach the initial target of a three-quarter MMR reduction by 2015 [5, 6]. Additional indicators related to unmet family planning needs and access by women to antenatal visits also remain below the set targets [4].

There is a compelling body of evidence highlighting the benefits of investing in reproductive health, demonstrating that spending in this sector is likely to have positive impacts not only on maternal and child morbidity and mortality, but also on general health, poverty reduction, and gender and social equity [7–11]. Increased government participation — through the removal of user fees and/or increased public revenue devoted to RH — has also shown to have a positive impact on family planning and maternal health service utilization [7, 12].

Maternal and reproductive health will remain key aspects of the post-MDG agenda [13, 14]. A recent report from the United Nations Secretary-General places a strong focus on maternal health — calling for an end to preventable maternal and child deaths — but also on broader gender concerns, including equal access to a full range of health services as well as sexual and reproductive health education. The report also calls for stronger policies to address demographic challenges and accelerate fertility decline [14].

In a context of shrinking donor commitment and global economic crisis, the mobilization and effective use of domestic resources are key elements to meeting MDG 5 by 2015, but they are also key to fulfilling future commitments in the area of maternal and reproductive health. The collection of relevant information on expenditure management and the allocation of funds to health — including to what extent this allocation reflects national priorities — is and will remain a key concern of local and international policymakers. Robust health resource tracking has been highlighted as a central element that serves a) to improve the efficient and equitable allocation of resources, b) to assess the adequacy of current funding levels, c) to promote accountability and transparency, and d) for use as an advocacy tool [15–17]. In the field of maternal and reproductive health, efforts to track both Overseas Development Assistance (ODA) and domestic resources have been central to the Countdown to 2015 initiative in 75 countries identified as ‘high-burden’ [18, 19]). The recent Global Financing Facility report further underlines how vigorous resource tracking systems can facilitate a transition towards more sustainable sources of domestic financing for maternal and child health activities [20].

As Burundi transitions from emergency aid to long-term development programs, there has been a growing focus on reproductive health as a way to contain demographic growth and reduce maternal and neonatal mortality. Since 2005, this interest has been systematically underlined in strategic documents released by the government. Burundi adopted ambitious maternal health and family planning targets, removed user fees for pregnant women and children, and introduced a performance-based mechanism to fund a basic healthcare package strongly oriented towards maternal health and family planning [21].

One would expect both the ambitious targets set by the government and the shift to a facility-based PBF (performance-based financing) approach to have translated into an increase in public funding for reproductive health activities, as this would send a clear signal to bilateral and multilateral partners about the importance of RH to the government. However, due to lacking information and weak financial systems, Burundi does not possess recent estimations for health expenditures; the latest National Health Account (NHA) dates to 2007. Furthermore, the country has never implemented a system to track RH expenses. This lack of available and accurate information hinders the development of a compelling argument for investing more in reproductive health activities.

The purpose of this study is to generate such evidence. We present estimates of public expenditure levels and trends in reproductive health activities in Burundi during 2010, 2011, and 2012, organized by a) the main reproductive health activities offered in the country, b) the main public financing agents, and c) health providers.

### Background

#### Development, health, and health system in Burundi

The health system in Burundi was vastly debilitated by its civil war, which ravaged the country for 13 years, ending in 2005 [22, 23]. Since then, Burundi remains a fragile state; GDP per capita was estimated at $102 in 2011 [22]. Life expectancy at birth is currently estimated at 50.9 years, four years below the regional average [24] (Table 1).

**Table 1:** Selected maternal and reproductive health, health, and development indicators: Burundi and sub-Saharan Africa, most recent years available

Indicator	Burundi	Sub-Saharan Africa average
***Health and development indicators***		
Population, 2012 (millions)^a^	9. 85	911.5
Urban population (% of total), 2012^a^	11	37
Annual population growth (%), 2012^a^	3.2	2.7
Population density (people per sq. km of land area), 2012^a^	384	39
Gross national income (GNI) per capita in PPP terms (constant 2005 Intl. $), 2012^a^	544	2,010
Population below income poverty line ($1.25 PPP per day) (%) 2012^b^	81.3	N/A
Human Development Index (HDI), 2012^b^	0.355	0.475
Life expectancy at birth, 2012^b^	50.9	54.9
Mean years of schooling (of adults) (years), 2010^b^	2.7	4.7
Adult literacy rate (% age 15 and older), 2012^b^	67.2	63
***Maternal and reproductive health indicators***		
Maternal mortality ratio per 1,000,000 live births, 2010^b^	800	475
Infant mortality rate (per 1,000 live births), 2010^b^	88	76
Under-five mortality rate (1,000 live births), 2010^b^	142	120
Total fertility rate per woman aged 15–49 (2012)^a^	6.1	5.1
Unmet need for contraception (% of married women ages 15–49), 2010 (2009 for regional average)^a^	32	25
Births attended by skilled health staff (%), 2010^a^	60	50
Contraceptive prevalence (% of women ages 15–49), 2010^a^	22	24
***Financial indicators (most recent year available)***		
Total health expenditure (% of GDP)	14^c^ (2007)	6.5^a^ (2012)
Health expenditure, public (% of GDP)	2.4^c^ (2007)	2.7^a^ (2012)
Public health expenditure (% of total health expenditure)	38^c^ (2007)	43.8^a^ (2012)
Health expenditure per capita (US$)	17.4^c^ (2007)	95^a^ (2012)

^a^World Bank Database (http://data.worldbank.org/region/SSA)

^b^Human Development Report 2013

^c^National Health Accounts of Burundi 2007, Ministry of Public Health and Fight Against HIV/AIDS, Burundi

Even so, the Burundian healthcare system is quickly evolving. Following the 2005 peace agreements, the focus of both the government and donors turned to addressing the basic health needs of the Burundian population, as most of the healthcare infrastructure had been destroyed in the civil conflict. Since then, bilateral and multilateral donors, such as USAID, the European Commission, JICA and the Dutch Minister of Foreign Trade and Development Cooperation have started to invest in long-term development programs [22]. Today, the healthcare system includes 45 health districts, covering 66 primary and secondary referral hospitals and 735 primary health care (PHC) clinics (423 public, 105 faith-based and 207 private clinics) [25]. Each PHC clinic offers a basic package of health services to the population, while primary and secondary referral hospitals offer emergency, hospitalization, laboratory, and diagnostic services. Finally, at the national level, seven third-tier institutions offer specialized care.

**Fig. 1:**
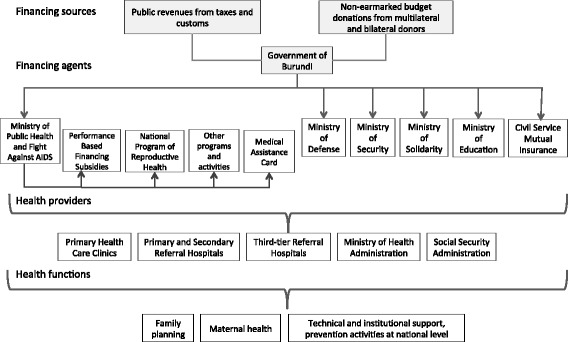
Financial Sources, Financial Agents, Health Providers and Health Functions related to public-sector financial contributions for reproductive health activities, Burundi

Overall, the health sector remains heavily dependent on external aid, which accounted for 40 % of total health expenditures in the country in 2007. An additional 43 % was covered by the private sector — mainly household or out-of-pocket expenditures (OOPs) — while the public sector accounted for the remaining 17 %. Part of the external aid is channeled through the public sector, which controls and manages 38 % of all health expenditures [26]. As part of the public sector, the Ministry of Public Health and Fight Against AIDS (MSPLS in French) acts as the primary financing agent. Other public agencies managing healthcare funds include the Ministry of National Defense and the Ministry of Public Security, which finance the healthcare of their respective employees, the Ministry of Higher Education and Research, which funds and manages the Teaching Hospital of Kamenge (CHUK), the Ministry of National Solidarity, Human and Gender Rights, which covers healthcare expenses for indigent populations, and Civil Service Mutual Insurance (MFP), the social security institution serving public employees throughout the country. Other ministries and public institutions also contribute funds to the healthcare system, though they simply make a financial contribution to the MFP on behalf of their employees. These actors account for the bulk of the public funding and provision of reproductive healthcare activities (Fig. 1).

### Reproductive health policies in Burundi

Since 2005, strategic documents in the public health sector have all underlined the essential role played by reproductive health, with a specific focus on maternal health and family planning (Table 2). The government, supported by its partners, increased training in reproductive and maternal health for healthcare staff, made additional investments in infrastructure and equipment, and increased funding for contraceptive supplies [25, 27–30]. In 2006, it also removed user fees for pregnant women and children under five who were using a basic package of services in health centers, primary, and secondary referral hospitals. This package is now financed through a performance-based mechanism (PBF) with support from the Ministry of Public Health, the World Bank, the European Union and other partners [21, 31]. In 2012, a total of $18.5 million was transferred to facilities using this payment mechanism, based on the volume of services provided for a defined set of indicators, [21]. Consequently, national statistics have shown an improvement in key reproductive health indicators [32]. Evidence also suggests that the PBF has led to an improvement in the probability of institutional deliveries, the use of modern family planning services, and the quality of most maternal and child care services [33, 34].

**Table 2:** Reproductive health in strategic health policies in Burundi since 2005

Name	Type	Period	Engagements taken related to RH	Adopted targets related to RH
Vision Burundi 2025	Multi-sectorial long-term strategic vision for Burundi	Adopted in 2011	Control demographic growth is one of the eight pillars of the Vision 2025 (Pillar 5). This will be done through implementing “an aggressive demographic policy” and putting a “particular stress” on family planning and reproductive health.	- Reduce the rate of population growth from 2.5 % to 2 % by 2025.
National Health Policy 2005-2015	Long-term plan for the health sector	Adopted in 2004 for the period 2005-2015	Two out of 12 strategic objectives relate to maternal health: #2 the reduction of maternal mortality and #5 reduction of low weight at birth	- Reduce by half the maternal mortality rate
				- Reduce by 1/3 the low birth weight rate
National Plan for Health Development I (PNDS I)	Operational five-year plan for the health sector	2006-2010	One of the four general objectives of the plan is to “reduce maternal and neonatal mortality”	- Reduce maternal mortality ratio by 30 % by 2010
				- Reduce pregnancy-related morbidity by 2010
National Plan for Health Development (PNDS II)	Operational five-year plan for the health sector	2011-2015	One of the three general objectives of the plan is to “contribute to maternal and neonatal mortality reduction by 2015”	- Reduce the maternal mortality ratio from 866 to 390 deaths per 100,000 live births by 2015
				- Reduce neonatal mortality rate by 50 % by 2015
IHP+ Burundi Country Compact	Contractual agreement between the government and technical partners for a better coordination and harmonization of health activities and funding	Adopted in 2008	The Country Compact aims at coordinating the work done by the government and technical and financial partners in the field of health. It follows the national framework adopted by the government and as such does not have specific objectives related to RH. However, two of its five results indicators are related to RH.	The indicators of results of the Country Compact include:- Percentage of institutional births
				- Number of Couple Years Protections (Family planning)

## Methods

In this study, we employed the reproductive health sub-account methodology (RHS), developed by the World Health Organization [35], which was adapted from the standard National Health Account methodology [36]. This accounting methodology is routinely used to track financial expenditures for the production, distribution, and consumption of reproductive health services and goods at the country level. We used this methodology to track expenditures for reproductive health services in Burundi in 2010, 2011, and 2012. This timeframe was selected because it corresponds to the transitional period between the first and second National Plan for Health Development (from 2006 to 2010 and 2011 to 2015, respectively). In addition, the study corresponds to the timeframe set by the 2005–2015 National Health Policy for the evaluation of the five-year, mid-term results [28].

### Operational definition of reproductive health

As defined by the World Health Organization, reproductive health should address “*the reproductive processes, functions and system at all stages of life.”* As such, reproductive health includes not only family planning or maternal and neonatal health, but in general all programs and policies allowing people to have a “*responsible, satisfying and safe sex life and (…) the capability to reproduce and the freedom to decide if, when and how often to do so*” [37].

The present study’s definition of reproductive health includes the two main components of reproductive health, implemented in Burundi:

- **Maternal health**, including activities such as prepartum and postpartum consultations, delivery attendance, vaccinations, malaria treatment for pregnant women, as well as the treatment of vaginal fistulas.
- **Family planning activities**, such as sexual education, the distribution of contraceptive methods, as well as vasectomies and tubal ligations.

Other reproductive health-related concerns, such as cancer, fertility treatments, sexual education for adolescents and young adults, and the prevention of gender-based violence were not included in the study, either because they are not offered as a public service in Burundi or because their implementation is not monitored at the national level.

RHS analysis includes four categories commonly used in health accounting [35]: financing agents, health providers, health functions, and health beneficiaries. Our study focuses on the impact of recent public policies on RH financial flows. Therefore, we decided to focus on the government as a financing agent (an institution receiving and managing funds to pay for health services) rather than focusing strictly on public sources of financing. Therefore, we included non-earmarked budget donations made directly to the Ministry of Finance by international donors in this analysis. Despite the fact that non-earmarked budget donations come from an external private source, the government maintains full discretion as to how to allocate these funds in its general budget. As such, we considered them to form part of public expenditures. These funds represented 16 % of the general government budget in 2012, 29 % in 2011, and 31 % in 2010 [38–40]. External donations earmarked for health expenses and managed by the Ministry of Health was not included, as the donor has already determined how it will be used.

Reproductive health expenditure was defined as the total amount of financial resources used to carry out the previously mentioned reproductive health activities. Only financial transactions paid out within Burundi during the years of analysis were included.

### Financing categories

**Public financing agents** were defined as the entities receiving funds and using them to pay for reproductive health services, products, and activities. In this study, six agencies were considered: the Ministry of Public Health and Fight Against AIDS, the Ministry of National Defense, the Ministry of Public Security, the Ministry of National Solidarity, Human and Gender Rights, the Ministry of National Education and the Civil Service Mutual Insurance.

**Healthcare providers** were defined as the entities delivering health services and the final recipients of healthcare funds. The following healthcare providers were included in this analysis: a) primary health care clinics, b) primary and secondary referral hospitals, c) third-tier referral hospitals, and d) the national-level administrative offices for the Ministry of Public Health and the Civil Service Mutual Insurance.

**Health functions** were defined as all activities developed by public institutions and actors with the objective of improving reproductive health among the Burundian population. The following activities were included: a) maternal health prevention and curative services, b) family planning activities and the provision of services, and c) administrative and leadership activities at the Ministry of Public Health and Fight Against AIDS and the MFP.

### Expenditure estimations and data analysis

We employed three different expenditure estimation strategies, depending on data availability:

1. Targeted funds earmarked for reproductive health

For all targeted funds clearly related to maternal and reproductive health, such as those allocated for the National Program for Reproductive Health, we included the total amount paid out for the year of study in our analysis.

2. Funds sent to health providers but not earmarked for reproductive health

Resources used by hospitals and clinics come from a mix of financial sources, including private out-of-pocket patient payments from public funding. For public funds provided by the Ministry of Public Health, the Civil Service Mutual Insurance, and other ministries that were sent to health providers but not directly earmarked for a specific health provider or for specific maternal and reproductive health activities (such as facility-level salaries, the building, or equipment expenditures), we used the following strategy:

- Estimations of total facility-level expenditures based on unit costs

In the first phase, we re-estimated the total health expenditures allocated to first-, second- and third-level healthcare providers, using a bottom-up approach based on unit costs. The estimated total health expenditures is, therefore, the product of the volume of services offered multiplied by the unit cost for each activity:

$$ \mathrm{T}={\displaystyle \sum \mathrm{V}\mathrm{h}\mathrm{s}*\mathrm{C}} $$

Where,

T is the total expenditure at the facility level (first-, second-, or third-level), including expenditures coming from both private and public sources,

Vhs is the volume or number of health services offered (including maternal health and family planning activities), and

C is the unit cost for each activity or service.

We obtained unit cost information from a 2012 MSH-Pathfinder micro-costing report [41]. Information on the volume of services produced was obtained from the 2010, 2011, and 2012 Annual Statistical Directories, which report the annual volume of services offered by provider and service type [32]. We classified these results into three categories: a) PHC clinics, b) primary and secondary referral hospitals and c) third-tier referral hospitals. Our results show that nearly half of all public health expenditures are allocated to PHC clinics, while an estimated 40 % is allocated to primary and secondary referral hospitals and roughly 16 % to 18 % is allocated to third-tier referral hospitals.

b. Estimations of facility-level expenditures on reproductive health activities

Next, we divided our estimated total expenditures for each provider across three main categories of health activities: a) maternal health, b) family planning, and c) other health activities not related to reproductive health. The allocation of funds depended on the set of services offered and the associated unit costs for each provider. We found that in 2012, approximately 28 % of total expenditures at PHC clinics are allocated to reproductive health activities. This proportion decreases to 13.3 % for primary and secondary referral hospitals, and to 10 % for third-tier referral hospitals (Table 3).

**Table 3:** Percentage of expenditures used for reproductive health activities by type of health provider in 2010-2012

Expenditures allocation across health activities (in %)	2010			2011			2012		
	PHC Clinics	Primary and Secondary Referral Hospitals	Third-tier referral hospitals	PHC Clinics	Primary and Secondary Referral Hospitals	Third-tier referral hospitals	PHC Clinics %	Primary and Secondary Referral Hospitals	Third-tier referral hospitals
Maternal health expenditure (A)	24.7	13.3	10.1	26.6	13.1	9.3	25.8	13.2	9.8
Family planning expenditure (B)	2.04	0.1	0.3	2.4	0.1	0.2	2.2	0.2	0.2
Total reproductive health expenditure (A + B)	26.8	13.4	10.4	28.9	13.3	9.5	28.0	13.3	10.0
Total expenditures in other health activities not related to RH (C)	73.2	86.6	89.6	71.1	86.7	90.5	72	86.7	90

Difference in total sums are due to rounding

We then used the distribution factors obtained through this calculation to parcel out non-targeted public expenditures to different providers for different services. This method was used to assign salaries for health providers, the cost of buildings and equipment paid out by the Ministry of Public Health, as well as expenditures from the Ministries of Defense, Security, Education and Solidarity and the Civil Service Mutual Insurance. We compared our estimates to the 2011 preliminary results on facility-level spending from the National Health Accounts and found comparable distribution among providers and among services (personal communication, planning and evaluation unit, Ministry of Public Health, October 2013).

3. National level administrative and leadership funds

Finally, with expenditures allocated for administrative and leadership activities at the national level (*e.g.* salaries and office expenses for the Ministry of Health’s national divisions), we used the following approach: first, we calculated a distribution factor by dividing the total direct public expenditures on reproductive health by the total direct public expenditures. We defined direct expenditures as all funds used directly for the provision of services (salaries, buildings, *etc.*). Then, we used this distribution factor to distribute public expenditures allocated to administrative activities at the national level.

We organized all the information into two matrices. The first included reproductive health expenditures by financing agent and by health functions. The second included reproductive health activities organized by health providers and health functions. Finally, we divided public expenditures on reproductive health by the estimated number of women of childbearing age and expected deliveries for the country [42] and compared these numbers with total public health expenditures (unpublished numbers provided by the MSPLS) and the national gross domestic product (GDP) of Burundi for each year [43]. We could not disaggregate the information further, by provinces or districts, due to lack of information at the sub-national level. All expenses were adjusted into constant 2012 Burundian Francs, using the inflation rate from the Central Bank of Burundi [43] and converted to international dollars (Int. USD), using the 2012 World Bank conversion factor [44]. The information was collected and processed using Microsoft Excel® 2011. The study included a secondary analysis of aggregated financial and health output data. It did not involve any human subject (human material or human data) and as such did not require ethical approval. Relevant public authorities in Burundi granted all the permissions necessary to access data not directly available to the general public.

## Results

Public expenditures on reproductive health in Burundi accounted for $41,163,141 international dollars in 2012. This amount marked an increase of 16 % from 2010 and of 2.6 % over 2011 levels. As a percentage of total public health spending, reproductive health spending also increased during the period, from 15 % in 2010, to 17 % in 2011, and 19 % in 2012. However, as a percentage of the country’s total GDP, public expenditures on reproductive health remained stable between 2010 and 2012 while the GDP grew at a faster rate during the same period, by 20 % between 2010 and 2012 (Table 4).

**Table 4:** Public expenditures on health and reproductive health: 2010–2012, Burundi (in thousands of 2012 International USD)

	2010	2011	2012
Total Public Expenditures - Reproductive Health	35,463 (15)	40,110 (17)	41,163 (19)
*Total Public Expenditures - Other Health Activities (excl. RH)*	202,871 (85)	196,556 (83)	178,995 (81)
Total Public Expenditures on Health	238,334	236,667	220,158
Public Reproductive Health Expenditures as a % of GDP	0.59 %	0.62 %	0.57 %

The numbers in parenthesis correspond to each component’s percentage of total public health expenditures

The Ministry of Public Health and Fight Against AIDS is by far the most important public financing agent for reproductive health activities in Burundi. In addition, the ministry’s share of total public RH spending increased during the period of study, from 73 % in 2010 to 76 % in 2012. The share spent by other public financial agents remained stable between 2010 and 2012, or even declined in real terms (Table 5). About half of all public expenditures on RH were paid out to PHC clinics, and, as expected, more than 70 % of these funds were dedicated to maternal health activities (Table 5).

**Table 5:** Public RH expenditures by financing agents, health providers and health functions: 2010–2012, Burundi (in thousands of 2012 International USD)

	2010	2011	2012
By Financing Agent			
Ministry of Public Health and Fight against AIDS	25,874 (73)	30,565 (76.2)	31,292 (76)
Civil Service Mutual Insurance (MFP)	7,453 (21)	7,515 (18.7)	7,909 (19.2)
Ministry of Higher Education and Research	576 (1.6)	517 (1.3)	582 (1.4)
Ministry of Public Security	697 (2)	649 (1.6)	587 (1.4)
Ministry of National Defense	818 (2.3)	809 (2)	747 (1.8)
Ministry of National Solidarity, Human and Gender Rights	45 (0.1)	55 (0.1)	46 (0.1)
By Health Provider			
Primary Health Care Clinics	18,239 (51.4)	18,776 (46.8)	21,599 (52.5)
Primary and Secondary Reference Hospitals	6,215 (17.5)	12,372 (30.8)	9,243 (22.5)
Tertiary Reference Hospitals	4,898 (13.8)	2,850 (7.1)	3,889 (9.4)
Ministry of Public Health and Fight Against AIDS Administration	3,131 (8.8)	3,216 (8)	3,383 (8.2)
Social Security Administration	2,981 (8.4)	2,896 (7.2)	3,048 (7.4)
By Health Function			
Family Planning	1,765 (5)	3,679 (9.2)	5,102 (12.4)
Maternal Health	27,586 (77.8)	30,319 (75.6)	29,630 (72)
Public Administration (except MFP)	3,131 (8.8)	3,216 (8)	3,383 (8.2)
Social Security Administration (MFP)	2,981 (8.4)	2,896 (7.2)	3,048 (7.4)

The numbers in parenthesis correspond to each component’s percentage of total public RH expenditures

According to the 2008 Burundian census, women of childbearing age represent 23.7 % of the total population and the number of deliveries each year was estimated to be equivalent to 5 % of the total population. The Burundian population grows by an average of 2.4 % each year [42]. Therefore, the average public RH spending per woman of childbearing age (defined by the Reproductive Health National Programme as women between the ages of 15 to 49 years old) was close to $20 international dollars for the year 2012. This amount remained relatively stable across the three years of study. RH expenditures per delivery, however, increased from $83.2 international dollars in 2010 to around $93 international dollars in 2011 and 2012.

As expected, the Ministry of Public Health’s funding is mainly allocated to the provision of health services. Salaries for peripheral-level, permanent administrative and health staff, as well as performance-based financing (PBF) subsidies, account for an estimated two-thirds of the Ministry’s total expenditures. A substantial increase in the share of PBF subsidies as a percentage of total expenditures took place between 2010 (16.5 %) and 2012 (46 %), demonstrating that this financing mechanism is becoming the dominant method used to reimburse health providers and cover facility-level expenses such as the salaries of temporary workers, supplies, and medicines (Table 6).

**Table 6:** RH expenditures from the Ministry of Public Health and Fight Against AIDS by main categories: 2010–2012, Burundi (in thousands of 2012 International USD)

RH Expenditures of the Ministry of Public Health and Fight Against AIDS By main categories	2010	2011	2012
Construction, rehabilitation and equipment for health facilities	6,350 (24.5)	6,323 (20.7)	1,166 (3.7)
Hospital endowment^a^	1,361 (5.3)	1,202 (3.9)	1,203 (3.8)
National Reproductive Health Programme	526 (2)	648 (2.1)	568 (1.8)
Performance-Based Financing subsidies for facilities^a^	4,281 (16.5)	9,851 (32.2)	14,404 (46)
Salaries and wages for peripheral-level employees (health facilities and local health agencies)	10,665 (41.2)	9 799 (32.1)	10,581 (33.8)
Central administration and central programs supporting RH activities	2,618 (10.1)	2,652 (8.7)	2,820 (9)
Medical Health Card^a^	73 (0.3)	90 (0.3)	75 (0.2)
Contraceptives^b^	N/A	N/A	474 (1.5)
Total	25,874	30,565	31,292

^a^Categories are not mutually exclusive. In particular, funds related to “Performance-Based Financing Subsidies”, “Medical Health Card” and “Hospital Endowment” can be used at facility level to finance equipment, supplies or salaries for temporary workers

^b^Expenditures linked to the procurement of contraceptives for 2011 were only paid out in 2012, and therefore are reported for this later year. No expenses linked to contraceptives were reported for 2010

## Discussion

This study sheds light on public reproductive health spending levels and trends in Burundi in recent years. First, our results show a steady increase in public expenditures on RH between 2010 and 2012, both in real terms and as a share of total public health expenditures. This is in line with the government’s expressed commitment to reproductive health in strategic documents and national plans. In addition, the distribution of public RH expenditures is aligned with the targets set by the government in official documents, which place a strong focus on maternal health and family planning. The majority of public RH funding continues to go to basic maternal health services, although the amount of funding dedicated to family planning nearly tripled between 2010 and 2012. Public RH funding also corresponds to the service delivery model promoted by the government; the bulk of public funding of RH is allocated to primary healthcare providers. Finally, our results confirm the growing impact of PBF on reproductive health expenditures between 2010 and 2012. In 2012, PBF subsidies represented 46 % of all RH financial flows managed by the Ministry of Health.

The lack of available and accurate data for previous years impedes an analysis of the long-term changes in public expenditures on RH in Burundi. However, these results can be compared with RH expenditure analyses from neighboring countries. In the early 2000s, RH sub-account analysis had shown a limited increase in terms of total and public RH financing. In Rwanda, despite a sharp increase in absolute public expenditures on RH between 2002 and 2006, RH spending represented only 5 % of total public health expenditures in 2006 [45]. A similar situation was observed in Malawi between 2002 and 2003 and 2004 and 2005 [46]. In the Democratic Republic of Congo (DRC), public expenditures on RH accounted for less than 1 % of total reproductive health expenditures in 2008 and 2009 [47]. Nevertheless, more recent results suggest that public institutions have increased funding for reproductive health. In Kenya, the government increased its RH funding by 55 % between the periods of 2005 and 2006 and 2009 and 2010. The relative contribution of the public sector to total RH funding also increased between these periods [48]. In Malawi, the share of RH spending in total public health expenditures jumped from 11.9 to 15.1 % between 2009 and 2010 and 2011 and 2012 [49]. At a global level, domestic funding for reproductive, maternal, neonatal, and child health grew by 21 % between 2010 and 2012 in the 75 countries classified as ‘high-burden’ as part of the Countdown to 2015 initiative [50]. The increased focus on reproductive health by the Burundian government during the period between 2010 and 2012 is in line with this recent trend.

However, this study also highlights the country’s enormous and unmet needs in the area of reproductive and maternal health. It shows that public expenditures per woman of childbearing age stagnated between 2010 and 2012 while public expenditures per delivery only slightly increased. This means that the total observed increase in public funding barely compensates for population growth. This increase in RH-related needs is not dissimilar to trends that are generally observed throughout the region. As highlighted by recent estimates, the development assistance for health (DAH) per disability-adjusted life year (DALY) for maternal, newborn, and child health in sub-Saharan Africa has remained stable over time, a trend explained by a sharp increase in DALYs coupled with a modest increase in DAH [51].

To address these needs, international donors and domestic institutions will be required to play complementary roles. Consolidated figures reveal a significant uptick in the share of DAH devoted to maternal, newborn, and child health development assistance, with a 17.7 % increase between 2010 and 2011 [51]. But as international financing for health is expected to stall, increased focus will be put on domestic funding in coming years [20, 50]. As the present study shows, local governments can ensure that public funds allocated to reproductive health are aligned with national priorities. This alignment between political priorities and funding flows can ultimately increase the efficiency of RH funding. The leadership of local institutions could also lead to the leverage of additional resources from international donors. In Burundi, the National Programme for Reproductive Health currently coordinates a general effort that aims to align public and donor funding with the country’s national RH strategy [31]. Shedding light on the financial involvement of the government in this sector can facilitate such coordination and ultimately ensure the success of RH policies.

National Health Accounts is an internationally-recognized methodology used to track expenditures across health systems. Its latest version allows for a complete distribution of expenditures across disease groups or services provided [36]. Such advanced analyses rely on regular resource tracking exercises and a well performing information system. In countries where limited financial information is available, the development of a sub-account system acts as an alternative, as it relies on ad-hoc estimation strategies based on available data. Although its use limits cross-country comparison in some ways, it can provide spending information for a specific service or sector that would otherwise not be estimated.

Estimations obtained through this study should be considered with the following informational limitations in mind. For some financing agents, available data were of low quality or insufficiently disaggregated, which may have limited our ability to correctly classify funding amounts. In addition, our team was unable to collect information about additional reproductive health activities, such as neonatal care or youth sexual education, creating uncertainty regarding the current levels of funding for these activities. Spending estimations were also limited to three years given that the team was unable to collect data from previous years. This has the effect of limiting the interpretation of results in search of long-term trends. Finally, external agencies financing reproductive health, such as bilateral and multilateral donors, or private financing from households or private firms, fell beyond the scope of this study due to lack of data availability and resource constraints. Our calculations thus represent only public expenditures; total levels of spending from all sources cannot be inferred from our findings.

## Conclusions

It is important to track public health expenditures, even in settings with poor information systems, not only to monitor stated governmental policies and political engagement, but also to ensure that international and domestic funding are aligned in their goals. As such, the estimations presented in this study provide important indicators about the levels of public spending on reproductive health in Burundi.

Our study also sheds light on how RHS methodology can be applied in countries with limited information systems. The post-2015 agenda will combine an increased focus on reproductive health activities with limited available development assistance for healthcare. In such a context, the efficiency and transparency of public RH expenditures will likely come into the spotlight. Strengthening financial tracking systems and developing their use according to local contexts will be key factors in predicting successful improvements to maternal and reproductive health. This is especially true for countries with limited national resources, such as Burundi.

## Abbreviations

CHUK: Teaching Hospital of Kamenge
DAH: Development Assistance for Health
DALY: Disability-adjusted life year
GDP: Gross domestic product
MDG: Millennium development goals
MFP: Civil Service Mutual Insurance (in French, Mutuelle de la Fonction Publique)
MSPLS: Ministry of Public Health and Fight Against Aids
NHA: National Health Account
ODA: Overseas development assistance
PBF: Performance-based financing
PHC: Primary health care
RH: Reproductive health
RHS: Reproductive health subaccount
